# Comparative full length genome sequence analysis of usutu virus isolates from Africa

**DOI:** 10.1186/1743-422X-10-217

**Published:** 2013-07-01

**Authors:** Birgit Nikolay, Anne Dupressoir, Cadhla Firth, Ousmane Faye, Cheikh S Boye, Mawlouth Diallo, Amadou A Sall

**Affiliations:** 1 Unité des arbovirus et virus de fièvres hémorragiques, Institut Pasteur de Dakar, Dakar, Senegal; 2 Université Cheikh Anta Diop Dakar, 24 Avenue Cheikh Anta Diop, Dakar, Senegal; 3 University Vienna, Dr. Bohr-Gasse 9/3, A-1030, Vienna, Austria; 4 CNRS UMR 8122, Institut Gustave Roussy, Villejuif 94805, France; 5 Center for Infection and Immunity, Mailman School of Public Health, Columbia University; New York, New York, United States of America; 6 Unité d’entomologie médicale, Institut Pasteur de Dakar, Dakar, Senegal

## Abstract

**Background:**

Usutu virus (USUV), a flavivirus belonging to the Japanese encephalitis serocomplex, was identified in South Africa in 1959 and reported for the first time in Europe in 2001. To date, full length genome sequences have been available only for the reference strain from South Africa and a single isolate from each of Austria, Hungary, and Italy.

**Methods:**

We sequenced four USUV isolates from Senegal and the Central African Republic (CAR) between 1974 and 2007 and compared the sequence data to USUV strains from Austria, Hungary, Italy, and South Africa using a Bayesian Markov chain Monte Carlo method. We further clarified the taxonomic status of a USUV strain isolated in CAR in 1969 and proposed earlier as a subtype of USUV due to an asymetric serological cross-reactivity with USUV reference strain.

**Results:**

A comparison of the four newly obtained USUV sequences with those from SouthAfrica_1959, Vienna_2001, Budapest_2005, and Italy_2009 revealed that they are all 96-99% and 99% similar at the nucleotide and amino acid levels, respectively. The phylogenetic relationships between these sequences indicated that a strain isolated in Senegal in 1993 is most closely related to the USUV strains detected in Europe. Analysis of a strain isolated from a human in CAR in 1981 (CAR_1981) revealed the presence of specific amino acid substitutions and a deletion in the 3′ noncoding region. This is the first fully sequenced human USUV isolate.

The putative USUV subtype, CAR_1969, was 81% and 94% identical at the nucleotide and amino acid levels, respectively, compared to the other USUV strains. Our phylogenetic analyses support the serological identification of CAR_1969 as a subtype of USUV.

**Conclusions:**

In this study, we investigate the genetic diversity of USUV in Africa and the phylogenetic relationship of isolates from Africa and Europe for the first time. The results suggest a low genetic diversity within USUV, the existence of a distinct USUV subtype strain, and support the hypothesis that USUV was introduced to Europe from Africa. Further sequencing and analysis of USUV isolates from other African countries would contribute to a better understanding of its genetic diversity and geographic distribution.

## Background

Usutu virus (USUV) is a member of the Japanese encephalitis serocomplex of flaviviruses that was isolated for the first time in 1959 in South Africa [1,2]. Since that time, USUV has been reported in several African countries [3] and was recognized for the first time in Europe in 2001 in association with the deaths of blackbirds (*Turdus merula*) and great grey owls (*Strix nebulosa*) in Austria [4]. Recently, USUV was identified in frozen samples from dead birds found in Italy in 1996, suggesting that an unrecognized introduction of USUV in Europe occurred prior to 2001 [5]. USUV has now been reported in several European countries and is thought to have established a transmission cycle involving local bird and mosquito species [6], similar to that suspected in Africa [7-15]. Although the natural transmission cycle of USUV involves mosquitoes primarily of the *Culex* genus and birds, two cases of human infection have been reported in the Central African Republic (CAR) and Burkina Faso [3], in addition to two recent cases of neuroinvasive infections in immunocompromised patients in Italy [16,17].

USUV is a positive sense single stranded RNA virus with a genome of approximately 11000 nucleotides (nt) with a type I cap structure and no poly(A) tail [18,19]. The genome consists of an open reading frame encoding a 3434 amino acid residue polyprotein that is cleaved into three structural proteins (core [C], membrane [PrM] and envelope [E]) that form the virus particle, and eight nonstructural proteins (NS1, NS2a, NS2b, NS3, NS4a, 2K, NS4b, and NS5) that perform essential functions for virus replication such as protease, polymerase, and methyltransferase activities [18]. Phylogenetic analysis of the nucleotide sequence of the 1959 isolate from South Africa [GenBank accession no. AY453412] resulted in the classification of USUV within the mosquito-borne cluster of flaviviruses, most closely related to Murray Valley encephalitis virus (MVEV) and Japanese encephalitis virus (JEV) [20,21]. At present, four full length genome sequences are available from South Africa, Austria [AY453411], Hungary [EF206350] and Italy [JF266698], and these are 97–99.9% and 99% similar at the nucleotide and amino acid levels, respectively [8,18,22]. Information on newly and previously sequenced USUV strains including host, location and time of isolation is summarized in Table 1. The pattern of observed sequence substitution suggests that it was not simply the South African strain that was introduced into Europe, therefore, it is likely that other USUV strains that are more closely related to the European isolates are circulating in Africa [18]. Despite the identification of USUV in Africa more than 40 years before its detection in Europe, full genome sequence is available from only one African isolate. Therefore, the genetic diversity of USUV in Africa remains unknown and the origin of this virus in Europe cannot be examined.

**Table 1:** Strains used for the comparative sequence analysis

**Strain**	**Isolate name**	**Geographic origin**	**Year**	**Host**	**Accession number**
SouthAfrica_1959	SAAR1776	South Africa	1959	*Cx. neavei*	AY453412
CAR_1969*	ArB1803	CAR	1969	*Cx. perfuscus*	KC754958
Kedougou_1974*	ArD19848	Kedougou (Senegal)	1974	*Cx. perfuscus*	KC754954
CAR_1981*	HB81P08	CAR	1981	Human	KC754955
Barkedji_1993*	ArD101291	Barkedji (Senegal)	1993	*Cx. gr. univittatus*	KC754956
Vienna_2001	Vienna_2001	Vienna (Austria)	2001	Blackbird	AY453411
MeiseH_2002		Austria	2002	Blue tit	JQ219843
Neunkirchen_2003	USU499-03	Neunkirchen (Austria)	2003	Nuthatch	EF078296
Strasshof_2003	USU450-03	Strasshof (Austria)	2003	Great tit	EF078295
Stegersbach_2003	USU502-03	Stegersbach (Austria)	2003	Blackbird	EF078297
Vienna_2003	USU281-03	Vienna (Austria)	2003	Blackbird	EF078294
Biberbach_2004	USU623-04	Biberbach (Austria)	2004	Blackbird	EF078300
Graz_2004	USU618-04	Graz (Austria)	2004	Blackbird	EF078299
Klosterneuburg_2004	USU338-04	Klosterneuburg (Austria)	2004	Blackbird	EF078298
Budapest_2005	Budapest	Budapest (Hungary)	2005	Blackbird	EF206350
Vienna_2005	USU589-05	Vienna (Austria)	2005	Blackbird	EF078301
	USU588-05				EF393679
	USU604-05				EF393680
	USU629-05				EF393681
Zurich_2006	Zurich 2006	Zurich (Switzerland)	2006	Blackbird	JX473238
Barkedji_2007*	ArD192495	Barkedji (Senegal)	2007	*Cx. neavei*	KC754957
Italy_2009	Italia 2009	Italy	2009	*Cx. pipiens*	JF266698
				Blackbird	
Piedmont_2009	USU173_09	Piedmont (Italy)	2009	*Cx. pipiens*	JN257983
	USU181_09				JN257984
Germany_2010	1477	Germany	2010	*Cx. pipiens*	JF330418
Giarole_2010	USU090_10	Giarole (Italy)	2010	*Cx. pipiens*	JN257982
CzechRepublic_2011	USUV-blackbird_Czechland_2011	Czech Republic	2011	Blackbird	JX236666
Mannheim_2011	BH65/11-02-03	Mannheim (Germany)	2011	Blackbird	HE599647

* newly sequenced strains.

In this study, we analyzed the sequences of USUV strains isolated in Senegal in 1974, 1993 and 2007 in the course of an entomological surveillance program. Additionally, as several cases of human USUV infections have been reported [4,16,17] but no sequencing of such strains has been done, we included an isolate from a human patient with symptoms including fever and rash from CAR in 1981. Analysis of the characteristics of the latter strain has the potential to reveal determinants of human virulence. We further investigated the taxonomic status of a serologically identified USUV subtype strain isolated in CAR in 1969 [23] to clarify whether it should be considered a distinct subtype or a new viral species.

## Results

### Sequence analysis of USUV strains circulating in Africa

The full genome sequences of the USUV strains Kedougou_1974 (ArD19848), CAR_1981 (HB81P08), Barkedji_1993 (ArD101291), and Barkedji_2007 (ArD192495) were 10800–10837 nt long and contained an ORF between nt positions 97 and 10401 in reference to SouthAfrica_1959 (SAAR1776). Conserved flavivirus motifs, already identified in the USUV strains from South Africa and Austria [18], were also found in the four newly sequenced isolates from Africa. Additionally, putative N-glycosylation sites (Asn-Xaa-Ser/Thr) could be identified at amino acid positions 118 and 154 of the E protein and are conserved among all USUV strains.

**Figure 1:**
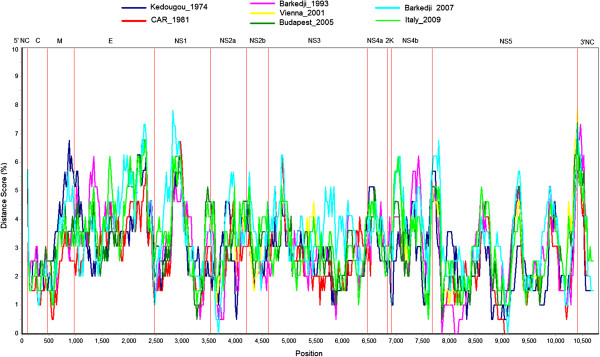
**Diversity plot of strain SouthAfrica_1959 and seven USUV strains.** The diversity in different regions of the genome was analyzed using the Simplot software and the Kimura 2-parameter model. The distance score is given in percent.

Multiple sequence alignment of the four newly sequenced USUV strains with the full length sequences from SouthAfrica_1959, Vienna_2001, Budapest_2005, and Italy_2009 revealed 96-99% and 99% similarity at the nt and amino acid levels, respectively. The nt sequence identity was 91-100% in the 5′ noncoding region, 96-99% in the ORF and 95-100% in the 3′ noncoding region. A diversity plot comparing all USUV sequences to the USUV isolate from South Africa indicated a homogenous distribution of sequence variability over the genome. A slight increase in diversity can be observed in the 3′ region of the M and E protein coding regions, the central region of the NS1 protein coding region and the 3′ noncoding region of the genome, while conserved regions were found primarily in the NS5 region (Figure 1).

**Figure 2:**
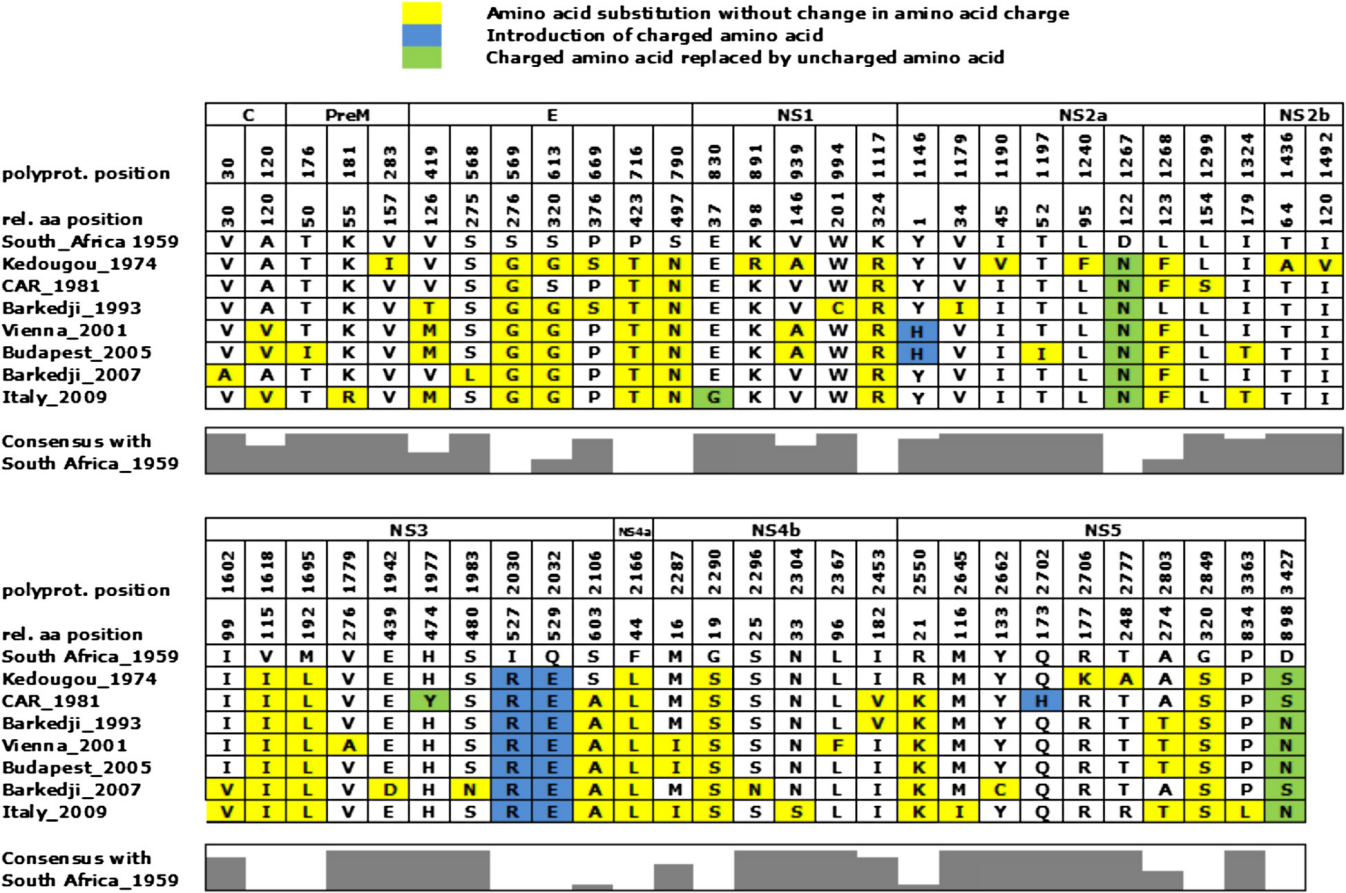
**Detailed amino acid sequence comparison of USUV strains.** Amino acid sequences of the four newly sequenced USUV strains (Kedougou_1974, CAR_1981, Barkedji_1993, Barkedji_2007) and the three isolates from Europe (Vienna_2001, Budapest_2005, Italy_2009) have been compared with the USUV reference strain SouthAfrica_1959 (SAAR1776). The amino acid positions of sequence differences in the polyprotein (polyprot. position) and each distinct protein are indicated (rel. aa position).

Comparison of the USUV amino acid sequences generated in this study with the SouthAfrica_1959 reference strain revealed an introduction of charged amino acids at position 1146 of the polyprotein in the strains isolated in Vienna and Budapest, at position 2030 and 2032 in all seven USUV strains, and at position 2702 in CAR_1981 (Figure 2). In contrast, a charged amino acid has been replaced by an uncharged amino acid at position 830 in Italy_2009, at positions 1267 and 3427 for all strains, and at position 1977 in CAR_1981. Interestingly, at positions 569, 716, 790, 1117, 1267, 1618, 1695, 2030, 2032, 2166, 2290 and 2849, all seven USUV strains differed from the reference strain. Specific amino acid substitutions in the strains from Europe can be found at positions 120 and 2287.

Of special interest is the strain CAR_1981, which was isolated from a patient with fever and rash. This strain differs from all other sequenced USUV strains at amino acid positions 1299, 1977, and 2702; the two latter mutations are associated with amino acid charge changes (Figure 2). Additionally, a 16 nt deletion in the 3′ noncoding region from nucleotide positions 10494 to 10510 was unique to this strain.

Positively selected sites in the USUV ORF could not be identified and the observed low mean dN/dS value (0.04) indicates the presence of strong purifying selection throughout the genome, as noted for other vector-borne RNA viruses [24].

**Figure 3:**
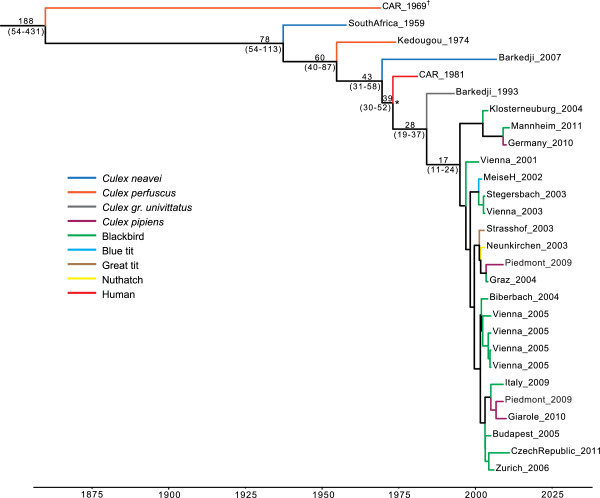
**MCC phylogeny of the E gene of USUV including the subtype (denoted with †), rooted using a relaxed molecular clock.** Branch tip times (x axis) reflect the dates of viral sampling. For each major node with Bayesian posterior probability (BPP) values >0.7, the corresponding mean and 95% HPD intervals for the age (years before present) are given, with the exception of the node marked with an asterisk (BPP = 0.6). Accession numbers and time-of sampling information for all sequences are given in Table 1. A color-code is used to reflect the different hosts from which USUV strains were isolated.

Bayesian phylogenetic analysis suggests that the South Africa_1959 strain shared a most recent common ancestor (MRCA) with those isolated in Senegal and CAR, as well as in Europe, 54 – 113 years before present (ybp) (Figure 3). Interestingly, Barkedji_2007 does not seem to have evolved directly from Barkedji_1993. Instead, these two strains last shared a common ancestor approximately 43 ybp (95% highest posterior density interval (HPD) = 31 – 58 ybp), and may represent distinct circulating strains. Of the viruses that have been sampled to date, Barkedji_1993 is the closest strain of African origin to the USUV isolates from Europe, sharing a MRCA with the European strains 19 – 37 ybp (Figure 3). The posterior mean rate of nucleotide substitution for the E gene of the USUV data set was estimated to be 1.37 × 10^-3^ subs/site/year (95% HPD = 0.290 – 2.56 × 10^-3^ subs/site/year).

### Comparison of USUV strains to CAR_1969 (putative USUV subtype)

The strain CAR_1969, isolated from *Cx. perfuscus* mosquitoes, has been serologically identified as a USUV subtype [23]. When using a complement fixation assay, the serum against the USUV reference strain SouthAfrica_1959 recognized SouthAfrica_1959 with a titer of 32, and the strain CAR_1969 with a titer of 8. Serum raised against CAR_1969 reacted against CAR_1969 with a titer of 64 and against SouthAfrica_1959 with a titer of 16, indicating heterogeneity and a close antigenic relationship between CAR_1969 and SouthAfrica_1959 [23].

**Figure 4:**
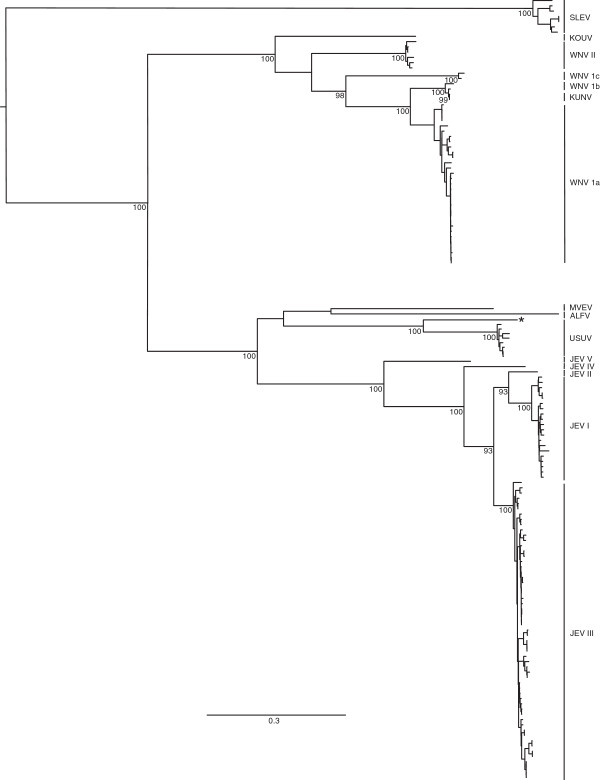
**ML phylogeny based on the polyprotein of the Japanese encephalitis (JE) group of flaviviruses.** The position of the Usutu subtype CAR_1969 is indicated by an asterisk (*). For clarity, bootstrap values ≥ 75% are given for those nodes leading to primary clades only. The tree is rooted based on the position of the JEV group relative to the rest of the Flaviviridae in a preliminary ML tree (data not shown). The branch lengths and scale bar are drawn to a scale of nucleotide substitutions per site.

A comparison of the genetic distances both within and between viruses in the Japanese encephalitis group demonstrates that the genetic distance within the entire USUV group (0.00-0.19 subs/site) does not exceed those estimated within JEV (0.01-0.21 subs/site) and West Nile virus (WNV) (0.00-0.22 subs/site), suggesting that CAR_1969 can be considered a subtype within USUV by this measure (Figure 4).

## Discussion

Although USUV has been reported in Africa for more than 50 years, only the SouthAfrica_1959 full genome sequence was available, and the genetic diversity of USUV in Africa was undescribed. Moreover, previous sequence comparisons of the SouthAfrica_1959 strain with isolates from Austria and Hungary indicated that the emergence of USUV in Europe could not be explained by an introduction from South Africa [18]. In this study, four USUV isolates from Senegal and CAR between 1974 and 2007 were sequenced and compared to the available full length genomes from South Africa, Austria, Hungary, and Italy. Despite their geographic distance and more than 48 years separating the dates of isolation, the genetic diversity of all USUV strains was low. The mean estimated time to MRCA of all sampled USUV strains was only 188 ybp (95% HPD = 54 to 431 ybp), a relatively recent estimate for the origin of USUV on the African continent. This is especially striking when compared to the TMRCA of yellow fever virus, for example, which has a mean estimated time to MRCA of more than 1000 ybp [25]. It is important to note, however, that the recent time to MRCA we estimated here for USUV represents only the genetic diversity of the sampled viruses, which is both geographically and temporally limited. Therefore, the isolation and sequence analysis of additional USUV strains from distinct geographic regions in Africa is likely to extend this estimate significantly.

Barkedji_2007 is the most recently isolated USUV strain; however, the MCC phylogeny suggests that this strain may be more distantly related to Barkedji_1993 and the strains isolated in Europe 20–47 years ago, then these strains are to each other (Figure 3). Therefore, genetically diverse USUV strains appear to be circulating nearly simulateously in the same geographic region. Interestingly, the strain sampled in Barkedji in 1993 was more closely related to the European USUV strains than to any other African virus. Taking into account the eight years difference between the dates of isolation, this finding supports the hypothesis that USUV was introduced into Europe from Africa. This introduction may have occurred through one of the ornithological natural parks in Africa as the one in the northern part of Senegal where many of the birds migrating between Europe and Africa stop for days or weeks [26]. Here, the opportunity would certainly exist for birds to become infected by circulating viruses and subsequently export them from Africa to Europe. Moreover, the limited genetic diversity of USUV in Europe might reflect a recent introduction of the virus, compared to the broader diversity observed in Africa, the likely origin of USUV. Alternatively, a narrower host or vector range in Europe could also result in the reduced genetic diversity observed. Nevertheless, three specific amino acid substitutions were observed in the isolates from Europe, which may have arisen through selection or as a result of the founder effect. Whether these mutations constitute adaptations to vector species abundant in Europe or influence the infectivity of host species remains to be investigated.

The importance of USUV as human pathogen and the mechanism of USUV virulence in people are poorly understood and only a few cases of human infection have been reported [3,16,17]. In this study, we sequenced a USUV strain isolated in 1981 in CAR from a patient with fever and rash [27]. Compared to all other USUV strains, three amino acid substitutions and a 16 nt deletion in the 3′ noncoding region were detected. However, the importance of these mutations for USUV virulence or replication in humans remains unclear. The comparison of CAR_1981 to other human isolates may help to identify virulence-determining sites in humans. Interestingly, the 3′ noncoding region is important for flavivirus replication and virulence determination, as the formation of secondary structures serves as cis-acting elements during RNA transcription [19]. The observed 16 nt deletion might alter these secondary structures and thereby influence virus infectivity in vertebrate or mosquito cells, resulting in a modified vertebrate host or vector range. These potential effects should be investigated in different cell culture systems and vector competence studies.

With the exception of CAR_1969, little genetic diversity was present between the sequenced USUV genomes. Therefore, the large number of substitutions observed in CAR_1969 may indicate that CAR_1969 should be considered a distinct viral species. Instead, we suggest that CAR_1969 should be considered a subtype of USUV, based on the genetic distance between all USUV strains including CAR_1969 (0.00-0.19 subs/site), which do not exceed those observed for other closely related viruses of the Japanese encephalitis group, namely WNV (0.00-0.22 subs/site) or JEV (0.01-0.21 subs/site). The designation of CAR_1969 as a subtype strain is further supported by the observed serological crossreactions between CAR_1969 and SouthAfrica_1959. It is important to note that the designation of viruses as distinct species is based not only on differences in genome sequence, but also differences in the biological properties or natural histories. Therefore, one can provisonnally classify CAR_1969 as an USUV subtype.

The results of this study indicate that sequence differences between strains isolated in Europe and Africa may be significant enough to reduce the accuracy of molecular diagnostic tests if not considered. Our results suggest that highly conserved regions among USUV strains suitable for primers design are found mainly in the NS5 region.

## Conclusions

This is the first study of the genetic diversity of USUV in Africa and the phylogenetic relationships of these strains to those identified in Europe. The results suggest that limited genetic diversity is present in the sampled USUV, and further strengthens the hypothesis that USUV was introduced into Europe from Africa. However, USUV isolations in Africa have been reported primarily from entomological surveillance programs and are therefore restricted to limited geographic areas. Surveying additional African countries for USUV may expand the known range of this virus and further contribute to our understanding of the genetic diversity and patterns of spread of USUV in Africa. This additional data will also be necessary to resolve the origin and timing of the introduction of USUV to Europe from Africa.

## Materials and methods

### Virus strains

The USUV strains sequenced in this study (Kedougou_1974, Barkedji_1993, Barkedji_2007, CAR_1969, CAR_1981) were provided by the CRORA (WHO Collaborating center for arboviruses and viral hemorrhagic fever viruses) of the Institut Pasteur de Dakar, either in lyophilized form or as brains of suckling mice intracerebrally inoculated with homogenate of ground mosquitoes (Kedougou_1974, Barkedji_1993, Barkedji_2007, CAR_1969) or human sera (CAR_1981). Information about the isolates analyzed in this study is summarized in Table 1.

### Virus amplification

The brains of suckling mice were homogenized in Leibovitz L-15 medium (GibcoBRL, Grand Island, NY, USA), centrifuged for 10 min at 8000 rpm at 4°C and the supernatants used for amplification. The lyophilized strains were suspended in L-15 medium. AP61 cells (*Aedes pseudoscutellaris*) were cultivated at 27°C in L-15 medium supplemented with 10% fetal bovine serum (FBS) (GibcoBRL, Grand Island, NY, USA), 10% of tryptose phophate (GibcoBRL, Grand Island, NY, USA), 1% penicillin/streptomycin (GibcoBRL, Grand Island, NY, USA) and 0.5% fungizone (GibcoBRL, Grand Island, NY, USA). Twenty five cm^2^ cell culture flasks (NUNC) of 80% confluent AP61 cells were inoculated with 100 μl supernatant of homogenized brains or suspension of lyophilized strains. After one hour of incubation at 27°C, 5 ml of AP61 medium supplemented with 5% FBS were added. Following an incubation at 27°C for 5 days, the infection was evaluated by immunofluorescence analysis using hyperimmue ascitic fluid specific for USUV as previously described [28]. The cell supernatants were stored at −80°C.

### Reverse transcriptase PCR

Viral RNA was extracted from cell culture supernatants using the QIAamp viral RNA extraction kit (Qiagen, Heiden, Germany) following the manufacturer’s instructions. RT-PCR was performed using either the AMV reverse transcription kit (Promega, Madison, USA) in combination with reverse primers (Additional files 1 and 2) or the Superscript II kit (Invitrogen, Carlsbad USA) combined with pdN6 random primers (Roche, Mannheim, Germany) following the manufacturer’s instructions.

### PCR

Amplifications were performed using the Go-Taq PCR kit (Promega, Madison, USA). The E, NS3 and NS5 regions were first amplified using flavivirus consensus primers as previously described (list of primers in Additional file 1) [21,29-31]. To obtain the full genome sequences, primers were designed in conserved regions of the USUV genome (list of primers in Additional file 2). The 5′ noncoding region of the genome was obtained using the 5′RACE kit (Invitrogen, Carlsbad, USA) with the primers 5primeR2 and 5primeR3, or 5primeR4 and 5primeR5 following the provider’s instructions (Additional file 2).

### Sequencing

PCR products were separated on 1% agarose gels in 1X TAE and extracted using the QIAquick Gel Extraction kit (Qiagen, Heiden, Germany) following the manufacturer’s instructions. Sequencing was performed by Beckman Coulter Genomics (Beckman Coulter Genomics, Takeley, UK).

### Sequence analysis

Putative N-glycosylation sites were identified using NetNGlyc1.1 [32]. Nucleotide and amino acid alignments of the USUV sequenced in this study with those available on GenBank were performed using ClustalW2 and modified manually (Table 1) [33]. Similarity plots were performed using the SIMPLOTv.1.3 software and the Kimura 2-parameter model [34].

### Selection pressure

To estimate the strength and nature of selection on individual codons and determine the overall nature of natural selection acting on the genome of USUV, the mean ratio of nonsynonymous to synonymous nucleotide substitutions (dN/dS) per site were computed using the single-likelihood ancestor counting (SLAC) method available in the Datamonkey web interface of the HY-PHY package, in combination with a general time-reversable (GTR) model of nucleotide substitution and an input neighbor-joining tree [35].

### Phylogenetic analysis

Maximum likelihood trees of all available USUV E gene sequences (with and without the subtype strain) were generated using PAUP*v4.0b and the GTR model of nucleotide substitution with an among-site rate heterogeneity parameter (gamma, G) with four rate categories, as determined by Modeltest 3.7 (Ntaxa=18, Nchar=1500) [36,37]. The clock-like behavior of each data set (with and without CAR_1969) was assessed by regressing the root-to-tip genetic distance inferred from the ML trees against time-of-sampling using the program Path-O-Gen v1.2 [38]. A Bayesian Markov chain Monte Carlo (MCMC) phylogeny of USUV incorporating time-of-sampling was estimated using BEAST v1.7.5 [39]. The analysis was performed using the SRD06 model of nucleotide substitution, a constant population size demographic model (the best-fit model, data not shown) and a relaxed molecular clock with an uncorrelated lognormal distribution of rates. Two independent MCMC runs were each performed for 100 million generations with subsampling every 10 000 generations. The runs were combined after removing a 10% burn-in from each. The maximum clade credibility tree was summarized using TreeAnnotator v1.7.5 available in the BEAST package.

An ML phylogeny of the complete polyprotein of USUV and representatives of all flaviviruses in the JEV group was created as above using a GTR+G model with invariant sites. The tree was rooted based on the phylogenetic position of the JEV group within the entire Flaviviridae family. A neighbor-joining bootstrap resampling analysis with 1000 replications was performed to assess nodal support using the ML substitution model.

### Nucleotide sequence accession numbers

The complete genomic sequences of strains CAR_1969, Kedougou_1974, CAR_1981, Barkedji_1993 and Barkedji_2007 were submitted to the GenBank database under the accession numbers KC754954-KC754958.

## Competing interests

The authors declare that they have no competing interests.

## Authors’ contributions

BN, AD, CSB, MD, AAS designed the study. BN, AD, CF and AAS performed the experiments and analyzed the data. BN, AD, CF, OF, MD, CSB and AAS wrote the manuscript. All authors read and approved the final manuscript.

## Supplementary Material

Additional file 1 Flavivirus consensus primers. Click here for file

Additional file 2 Primers used for the partial amplification of USUV genomes. Click here for file
