# Supplementary material for: Comparative full length genome sequence analysis of usutu virus isolates from Africa
Source: Virol J. 2013 Jul 1;10:217. doi: 10.1186/1743-422X-10-217 (PMC3716710; doi:10.1186/1743-422X-10-217)
Supplement: Additional file 1 — Flavivirus consensus primers. [file 1743-422X-10-217-S1.doc]

| **Name** | **Sequence (5’-3’)** | **Position** | **Reference** |
| --- | --- | --- | --- |
| Unifor  Unirev | TGGGGNAAYSRNTGYGGNYTNTTYGG  CCNCCHRNNGANCCRAARTCCCA | 1276  2270 | [29] |
| FU1  FD3 | TACAACATGATGGGVAARAGWGARAA  ARCATGTCTTCYGTBGTCATCCA | 9043  10127 | [20] |
| EMF1  VD8 | TGGATGACGACGGAAGACATG  GGGTCTCCTCTAACCTCTAGT | 10105  10853 | [30] |
| NS3X1  NS3X2 | YIRTIGGIYTITAYGGIWWYGG  RTTIGCICCCATYTCISHDATRTCIG | 5040  5856 | [28] |

Additional file 1
