# Supplementary material for: Comparative full length genome sequence analysis of usutu virus isolates from Africa
Source: Virol J. 2013 Jul 1;10:217. doi: 10.1186/1743-422X-10-217 (PMC3716710; doi:10.1186/1743-422X-10-217)
Supplement: Additional file 2 — Primers used for the partial amplification of USUV genomes. [file 1743-422X-10-217-S2.doc]

| **Name** | **Sequence 5’ -3 ‘** | **Position** |
| --- | --- | --- |
| 5prime R2 | CCCGATTGTTAACCACGTTG | 391 |
| 5prime R3 | GGTGTTTCATTGCCGTGGTC | 337 |
| 5prime R4 | ATTGCCGTGGTCTTGTTGAT | 329 |
| 5prime R5 | TCATGACTTTCCCCTGGAAG | 508 |
| UsuF1 | CCAGTGCGATTTGTGCTG | 223 |
| UsuR1 | TGCCCACTGACATGATGTA | 1598 |
| FlaviNC5’ | AGWHGTTHRYCTRYGTGRRCY | 1 |
| 382 _EnvR1 | GCCTGTGATGCTCCTAGTTGTG | 1466 |
| UsuF2 | CATGGCAACTATTCTTCACAA | 1429 |
| UsuR2 | ACCCAAAGTCCCACGCTG | 2261 |
| 382_EnvF1 | GCGAAAGTGCTGGTTGAGATG | 2077 |
| 13_NS1R1 | ATCGCACATCCCGAGTCTGC | 2492 |
| UsuF8 | CTGCAGTACACGGGATCTGA | 1951 |
| UsuR8 | CTCTGCGGTGCTGATTTGTA | 2783 |
| UsuF3 | GATCACCCAGGGTCTAATG | 2349 |
| UsuR3 | AGCCGGAACAGTCAATCT | 3639 |
| 382_EnvF2 | TTCGGTCGGAGGGATTTTCA | 2259 |
| 382_NS3R2 | CCCCAGAATGTAACGGCTCA | 4701 |
| UsuF4 | TGTTGGTATGGAATGGAGATA | 3460 |
| UsuR4 | GGCTCATGATACGGTATACTC | 4687 |
| UsuF4-2 | CCATGAAGCATGATGAGACG | 3485 |
| UsuR4-2 | GCCACAGGGTGTGAAGAACT | 4756 |
| 13_NS3F1 | GATGGTGACTTCCACCTTAT | 4456 |
| 382_NS3R1 | TATCCTCCTGGTCTTTCCCG | 5218 |
| UsuF5 | CAATACTGGACAAGAATGGC | 5018 |
| UsuR5 | GCATTTGGGATACTCCGT | 5799 |
| 13_NS3F2 | CAGACATACAAGCTGAAGTGC | 5606 |
| 382_NS3R3 | CAAAAGCCTCTCTGGTTTTG | 6535 |
| UsuF6 | AGTCGAAATTGTCACCCG | 6342 |
| UsuR6 | CAACCACCTTGCCAATTG | 7918 |
| UsuF6-2 | TGTGGCTTGCCTACAAAGTG | 6239 |
| UsuR6-2 | ACCTTGCCAATTGGTTTGAC | 7913 |
| UsuF7 | CCTGAAGTACAGGAAAGAGGC | 7755 |
| 382_NS5R1 | CCGTGTCATCTGCGTACATT | 9295 |
| FUusu | TGGGCTTTCTGAATGAGGAC | 9155 |
| FDusu | TGTCATCCATTCACCTGTGG | 10113 |
| UsuF10 | AGAGTTTGGCAAAGCGAAAG | 9078 |
| UsuR10 | ATGTTCCTGCCCAATCACTC | 9591 |
| UsuF11 | AGACAACTGGCCAGAGCAAT | 9373 |
| UsuR11 | GTTTATGGCCGCGTAGATGT | 10302 |
| 382_NS5F1 | TACGCTCAGATGTGGCTCTTG | 9970 |
| VDusu | CTCCTACAGCTTCGGAAACG | 10916 |

**Additional file 2**
